# Variations in the Potential Suitable Habitats of Different Populations of *Hippuris vulgaris*, a Species with Cross-Altitude Distribution

**DOI:** 10.3390/plants14121798

**Published:** 2025-06-12

**Authors:** Junping Xu, Qiang Liu, Jiayu Ma, Weike Yan, Huijun Guo, Kun Tian

**Affiliations:** 1 Forestry College, Southwest Forestry University, Kunming 650224, China; xujunping79@163.com; 2 Yunnan Key Laboratory of Plateau Wetland Conservation, Restoration and Ecological Services, Southwest Forestry University, Kunming 650224, China; liuq03@mail.kiz.ac.cn; 3 National Plateau Wetlands Research Center, College of Ecology and Environment, Southwest Forestry University, Kunming 650224, China; 15135658993@163.com; 4 Sichuan Forestry and Grassland Investigation and Planning Institute, Chengdu 610081, China; ywk445027709@163.com

**Keywords:** climate change, different population, *Hippuris vulgaris*, maximum entropy model, suitable habitat

## Abstract

Understanding the variations in the potential suitable habitats of different populations of the same species is crucial for targeted biodiversity conservation and ecosystem management in specific regions. For widely distributed species, the impact of climate change on the suitable habitats of different populations may vary. However, research in this area is currently insufficient. *Hippuris vulgaris* is an aquatic species widely distributed across the Northern Hemisphere, with an altitudinal range from 0 to 5000 m, known for its high ecological and medicinal significance. In this study, we employed a MaxEnt model to simulate the current and future suitable habitats of *H. vulgaris* through constructing high-altitude, low-altitude, and integrated distribution models. The results indicated that bio3 (isothermality), bio1 (annual mean temperature), and bio19 (precipitation of coldest quarter) significantly influenced the distribution of high-altitude populations of *H. vulgaris*, whereas in low-altitude areas, bio3, bio9 (mean temperature of driest quarter), and bio13 (precipitation of wettest month) were the main influencing factors, and for integrated distribution populations, bio1, bio13, and bio19 were the main factors. The suitable habitat area for high-altitude populations of *H. vulgaris* will be increased by 19.66% in the 2050s but decrease by 47.75% in the 2070s. The suitable habitat area for low-altitude populations will be increased by 99.71% in the 2050s and by 13.29% in the 2070s. Our findings showed that the key bioclimatic variables and suitable values influencing the distribution of *H. vulgaris* populations in high- and low-altitude regions differed, and changes in the suitable habitats for high- and low-altitude populations showed completely opposite trends under climate change, with migration directions extending towards higher altitudes and higher latitudes, respectively.

## 1. Introduction

Climate is a primary determinant of species distribution, with changes in species distribution patterns directly reflecting climate change [1,2]. Currently, numerous studies have employed the MaxEnt (Maximum Entropy) model to predict future species distributions and niche dynamics across diverse taxa and ecosystems [3,4], but research on differences in distribution patterns of the same species across different regions remains limited. Under the climate change scenario, populations of species with a wide ecological niche range at different altitudes may show similar differences among different species, and it is necessary to evaluate them separately in each region [5]. Species distributed at high altitudes are more sensitive to climate change than those at lower altitudes [6,7], and their distribution range may shrink in the future, while migrating to higher altitudes faster [8]. Under climate change, the suitable habitat area of *Carex moorcroftii* and *Pegaeophyton scapiflorum* in the Tibetan Plateau has significantly decreased, with an increase in altitude [9,10]. In contrast, species in low-altitude regions may experience an expansion in their distribution range, albeit at a relatively slower migration rate [11,12]. Research on three congeneric species of Bergenia distributed at different altitudes found that the upslope migration rate of the high-altitude species *Bergenia purpurascens* was much higher than that of the low-altitude species *Bergenia ciliata* [13]. This indicates that high-altitude plants are more susceptible to climate change than their low-altitude counterparts. For species with broad distributions across altitudes, whether the distribution pattern differences between high-altitude and low-altitude populations are similar to those between high-altitude and low-altitude species remains unclear. Based on the principle of population divergence, delineated ecoregions can be used as training units within the model. This allows a species range to be divided into different populations, which, in turn, facilitates the subsequent tracking of different populations under climate change projections [14]. Investigating the potential habitat changes of populations of the same species in different regions is extremely important for the development of biodiversity conservation and ecosystem management strategies.

*H. vulgaris* is a perennial herbaceous plant that belongs to the genus *Hippuris* under the Plantaginaceae family [15], widely distributed along the margins of ponds, lakes, streams, and ditches in the Northern Hemisphere from an altitude of 0 to 5000 m [16]. Meanwhile, as one of the most cold-tolerant emergent aquatic plants globally, it forms monodominant communities in plateau wetlands at an altitude above 3000 [17,18], playing an extremely important role in maintaining the structural stability and functional integrity of these ecosystems [19]. Additionally, it is a key component of Tibetan and Mongolian traditional medicines, used as an endemic Chinese medicine to cure tuberculosis and cough [20]. As a species with a broad altitudinal distribution, *H. vulgaris* exhibits a strong adaptability to environmental changes [21], which also implies the existence of intraspecific trait variation across different altitudinal habitats [22]. This variation may lead to differences in the distribution patterns of *H. vulgaris* populations at different altitudes under climate change. Therefore, understanding these differences is crucial for targeted species conservation and ecosystem restoration in specific regions.

Species distribution modeling (SDM) is employed as the primary method to simulate suitable habitat areas and predict distribution changes based on environmental variables [4]. The maximum entropy model (MaxEnt), one of the most frequently used niche models, uses maximum entropy to calculate the potential geographical distributions of species, with the advantages of small distortion, good stability, and simple operation. In addition, because the MaxEnt model can accurately predict the future potential geographical distribution areas of species and visualize the spatial pattern changes in the suitable habitat areas of species, it has been widely used to explore the potential distribution areas of species [23]. In this study, we utilized the MaxEnt (maximum entropy) model to construct the following three population distribution models for *H. vulgaris*: a high-altitude distribution model, a low-altitude distribution model, and an integrated distribution model, thereby simulating suitable habitats for *H. vulgaris* under current conditions and for future periods based on occurrence records and climatic variables. Specifically, we aimed to (1) identify the most important bioclimatic variables affecting the distribution of *H. vulgaris* and (2) determine variations in the distribution and change trends of potential suitable habitats for *H. vulgaris* in different altitudinal models under climate change.

## 2. Materials and Methods

### 2.1. Collection and Processing of Distribution Records

**Figure 1:**
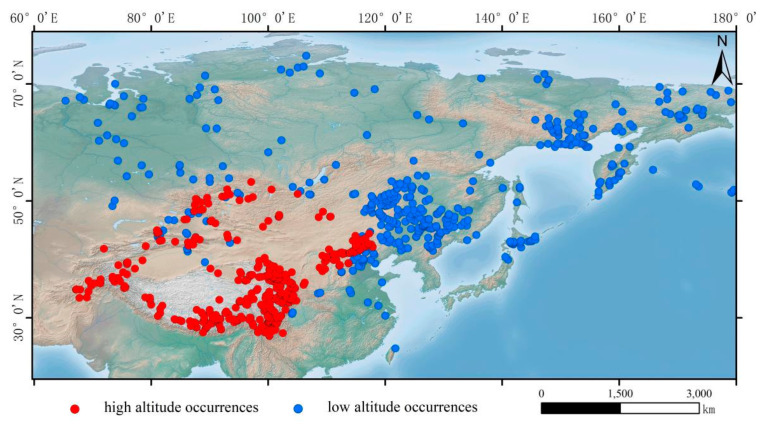
Occurrences of *H. vulgaris* in Asia.

The distribution data for *H. vulgaris* were primarily obtained from the Global Biodiversity Information Facility (GBIF, http://www.gbif.org/, accessed on 27 November 2023), the Chinese Virtual Herbarium (CVH, http://www.cvh.ac.cn/, accessed on 28 November 2023) and the iPlant Species Information System (iPlant, http://www.iplant.cn/, accessed on 30 November 2023), supplemented with relevant domestic and international research and field investigations. We verified the occurrence records of *H. vulgaris*, and records not located in Asia were excluded. To eliminate sampling bias, the obtained distribution points were organized into a CSV file format by species name, longitude, and latitude and then imported into ArcMap 10.6 to overlay with the environmental variables used. Distribution data outside the range of the environmental variables were removed. To minimize the interference of spatial autocorrelation in model construction, the distribution data were filtered using the Wallace platform in R 4.2.2 [24], ensuring that only one occurrence point was retained within each 2.5 min × 2.5 min grid cell [25,26]. Ultimately, 800 valid distribution records of *H. vulgaris* across the Asian continent were obtained. Based on elevation, distribution points above 1000 m were defined as high-altitude occurrences, resulting in 396 valid points, while those below 1000 m were defined as low-altitude occurrences, yielding 404 valid points (Figure 1).

### 2.2. Environmental Variable Selection

In this study, 19 bioclimatic variables from the WorldClim database (https://www.worldclim.org/) were used for model analysis (Table 1) at a resolution of 2.5 arc minutes. The bioclimatic variables for the future periods were available for 2041–2060 (the 2050s) and 2061–2080 (the 2070s). The Global Change model (BCC-CSM 2-MR) climate system model in the Sixth International Coupled Model Comparison Plan (CMIP 6) was selected, including shared socioeconomic pathways (SSPs), representative concentration pathways (RCPs), and the general development pathway SSP2-RCP4.5 (SSP245), namely the combined scenario of moderate radiative forcing and moderate social vulnerability. This scenario assumes that global CO_2_ emissions will remain at the current levels until mid-century, then begin to decline, but will not reach zero by the end of the century. Concurrently, global economic development follows historical trends without significant changes, resulting in a temperature increase of 2.7 °C by the end of the century [27].

The environmental data were first transformed into the ASC format required for the MaxEnt model. To avoid multicollinearity caused by the 19 bioclimatic variables and overfitting the model, multiple linear correlation analysis was conducted on the environmental variables used by the model, and the correlation coefficient between environmental variables combined with the contribution rate of environmental variables was used as the basis for screening [28,29]. The “Band Collection Statistics” tool in ArcMap 10.6 was used to analyze the correlation of the 19 environmental variables, and an absolute value of the correlation coefficient |r| of > 0.8 was selected as the standard for a strong correlation between environmental variables. Then, the 19 environmental variables were imported into the MaxEnt model for the trial run, and environmental variables with higher contribution rates were retained when the absolute value of the correlation coefficient |r| was > 0.8. By these methods, eight bioclimatic variables—bio1, bio2, bio3, bio7, bio8, bio13, bio15, and bio19—were selected for use in the integrated distribution model and the high-altitude distribution model, while bio2, bio3, bio7, bio8, bio9, bio13, bio15, and bio17 were selected for use in the low-altitude distribution model.

### 2.3. Model Optimization and Accuracy Evaluation

To enhance the accuracy of the MaxEnt model predictions, the distribution model of *H. vulgaris* was optimized using the Wallace Species Distribution Platform in R 4.2.2. The optimal Regularization Multiplier (RM) and Feature Combination (FC) were selected. In Wallace, the range of RM was set from 0.5 to 4.0 with an interval of 0.5. The FC options included H (hinge and fragmented), L (linear), LQ (linear and quadratic), LQH (linear, quadratic, and hinge), and LQHP (linear, quadratic, hinge, and product). Delta. AICc is a model evaluation metric, and the model with the lowest delta. AICc value among the candidate models was determined as the optimal model [30,31]. Based on the lowest delta. AICc values from the Wallace calculations, the optimal RM and FC were chosen for the following three models: for the high-altitude distribution model, RM = 3 and FC = LQHP; for the low-altitude distribution model, RM = 0.5 and FC = LQ; and for the integrated distribution model, RM = 3.5 and FC = LQHP.

Other parameter settings were as follows: model training was performed using 75% of the distribution data, and the remaining 25% of the data were used for model testing. The maximum number of background points was set to 10,000. The running process was repeated 10 times using bootstrap as the replicated run type, and the average result was taken as the final output. The replication mode was set to choose cross-validation. The maximum number of iterations was set to 1000. The 10th percentile training presence threshold rule was selected to delineate suitable and unsuitable habitats. Other settings were left at their default values.

The accuracy of the model was evaluated using the area under the receiver operating characteristic (ROC) curve (AUC), which ranges from 0 to 1. We evaluated the model’s accuracy using the average test AUC value from 10 repeated runs. In general, an AUC of >0.90 is considered excellent, between 0.80 and 0.90 is good, between 0.70 and 0.80 is fair, and <0.70 is poor [32,33].

### 2.4. Classification of Suitable Habitat and Centroid Distribution

The threshold for delineating unsuitable and suitable habitats was set at the 10th percentile training presence logistic mean value (0.3) after 10 runs. To facilitate display, we categorized the distribution maps for different periods into different levels, following the classification system for assessing likelihood in the Sixth IPCC Report. We designated regions with a species occurrence probability of 0.7–1 as high-suitability areas, 0.5–0.7 as medium-suitability areas, 0.3–0.5 as low-suitability areas, and below 0.3 as unsuitable areas [34,35]. For each distribution model, the suitable habitat distribution areas were calculated for different periods. Reclassification was then performed to generate change distribution maps and centroid migration maps for these periods.

## 3. Results

### 3.1. Model Validation, Dominant Bioclimatic Variables, and Response Curves

The AUC values for the high-altitude distribution model, low-altitude distribution model, and integrated distribution model were 0.919, 0.797, and 0.799, respectively, indicating excellent or good levels and suggesting that the models were effective in simulating suitable habitats for *H. vulgaris*.

**Figure 2:**
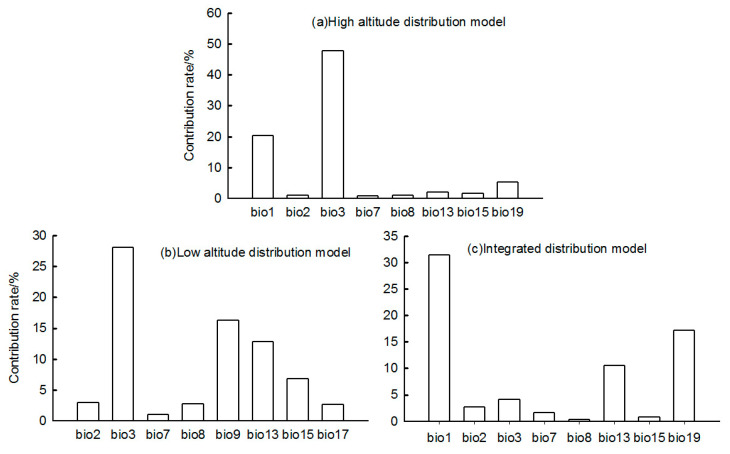
Contribution rates of bioclimatic variables.

**Figure 3:**
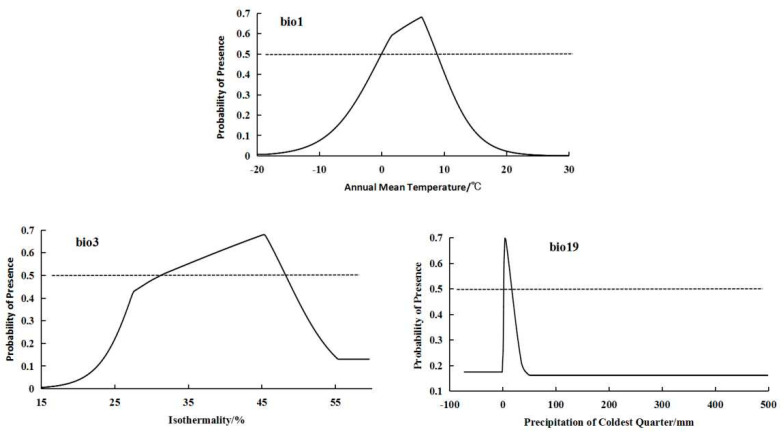
Response curves of important bioclimatic variables in high-altitude distribution model.

For the high-altitude distributed population, the bioclimatic variables with significant impacts on distribution were bio3 (isothermality), bio1 (annual mean temperature), and bio19 (precipitation of coldest quarter) (Figure 2). The high-altitude-distributed population showed a relatively suitable range of isothermality from 31.25 to 41.8, a suitable annual mean temperature range from −0.02 to 8.84 °C, and a suitable precipitation of coldest quarter range from 2.32 to 16.24 mm. The response curves of the high-altitude-distributed population to these three bioclimatic variables all exhibited a “unimodal” pattern, initially increasing and then decreasing (Figure 3).

**Figure 4:**
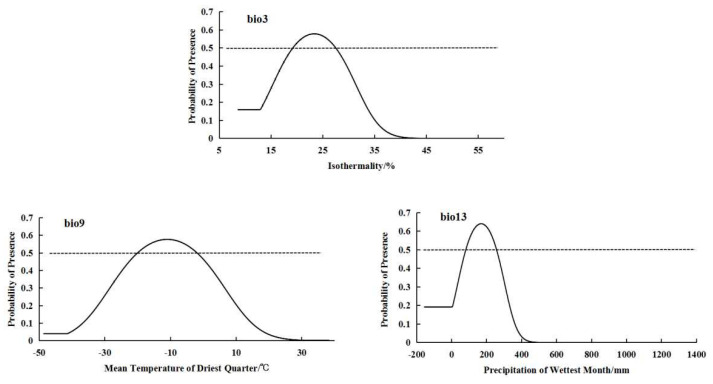
Response curves of important bioclimatic variables in low-altitude distribution model.

For the low-altitude-distributed population, the bioclimatic variables with significant impacts on distribution were bio3 (isothermality), bio9 (mean temperature of driest quarter), and bio13 (precipitation of wettest month) (Figure 2). The low-altitude-distributed population showed a relatively suitable range of isothermality from 19.25 to 27.47, a suitable mean temperature of driest quarter range from −20.03 to −1.95 °C, and a suitable precipitation of wettest month range from 83.32 to 256.67 mm. The response curves of the low-altitude-distributed population to these three bioclimatic variables also exhibited a “unimodal” pattern, initially increasing and then decreasing (Figure 4).

**Figure 5:**
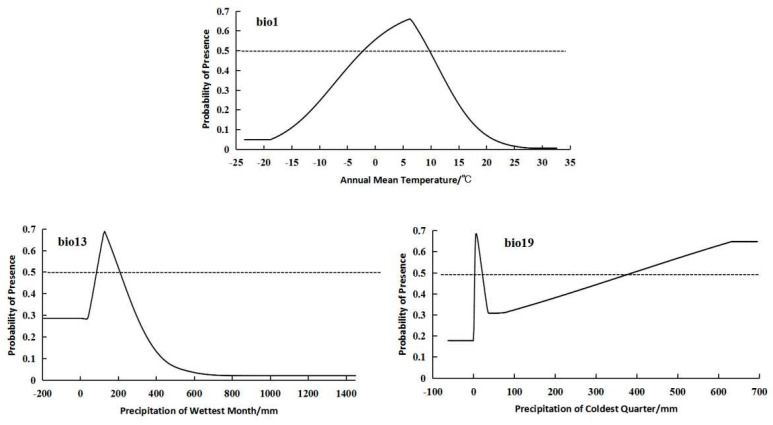
Response curves of important bioclimatic variables in integrated distribution model.

For the integrated distribution population, the bioclimatic variables with significant impacts on distribution were bio1 (annual mean temperature), bio13 (precipitation of wettest month), and bio19 (precipitation of coldest quarter) (Figure 2). The integrated distribution population showed a relatively suitable annual mean temperature range from −2.12 to 9.72 °C, a suitable precipitation of wettest month range from 87.92 to 207.23 mm, and a suitable precipitation of coldest quarter range from 2.78 to 20.224 mm and from 389.56 to 695.2 mm (Figure 5).

### 3.2. Distribution and Trends of Potential Suitable Habitats

**Figure 6:**
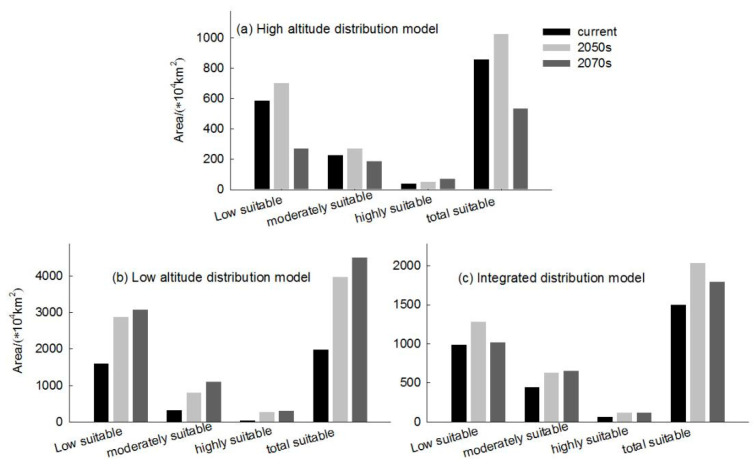
Suitable areas of *H. vulgaris* in different periods.

**Figure 7:**
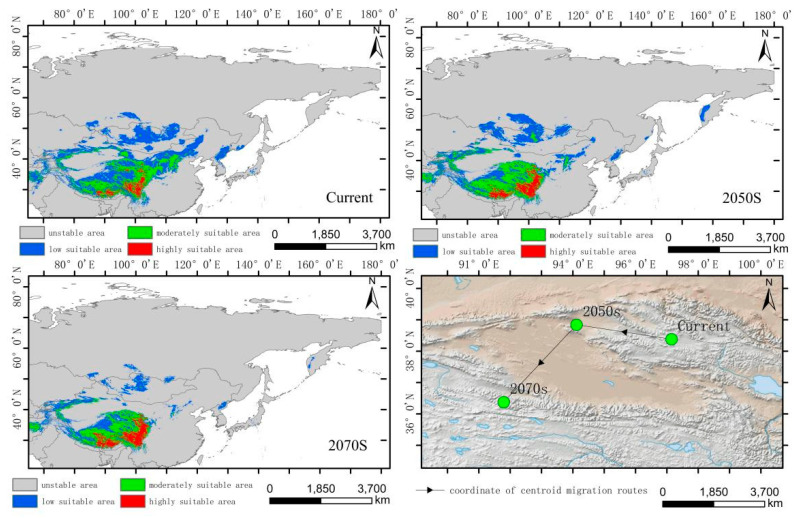
Distribution of suitable areas and centroid transfer of *H. vulgaris* in different periods in high-altitude distribution model.

**Figure 8:**
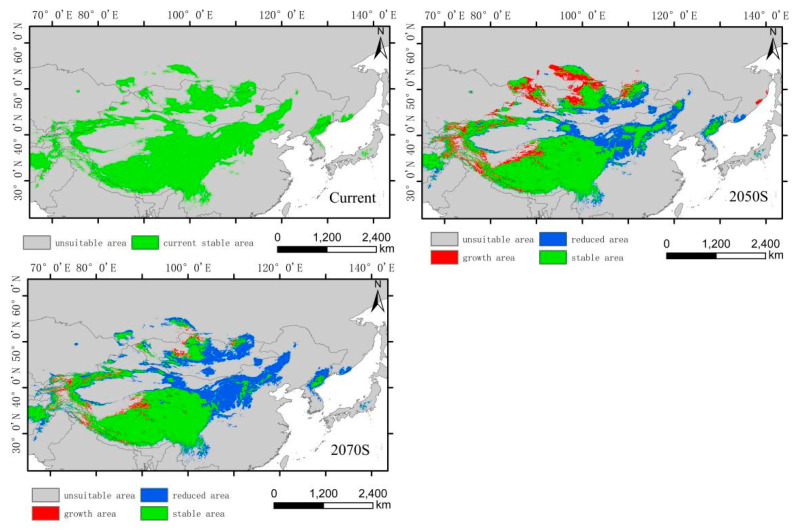
Dynamic changes in suitable area of *H. vulgaris* in different periods in high-altitude distribution model.

The simulated results show that the main suitable habitats for the high-altitude-distributed *H. vulgaris* are primarily located in the Tibetan Plateau, Mongolian Plateau, Inner Mongolia Plateau, Pamir Plateau, and the Tianshan and Altai Mountains under current conditions. Comparing the suitable habitat areas and distributions between the current and future periods reveals that the suitable habitat area for plateau-distributed *H. vulgaris* increases by 19.66% in the 2050s but decreases by 47.75% in the 2070s (Figure 6). The increased regions are primarily located in the southwestern and northwestern parts of the Tibetan Plateau, Pamir Plateau, and northwestern Mongolian Plateau. In the 2070s, the suitable habitat area decreases significantly, with a sharp contraction in the Mongolian Plateau, Inner Mongolia Plateau, and at the southern edge of the Hengduan Mountains. The highly suitable habitat area increases to some extent in both future periods, primarily concentrated along the southern edge of the Tibetan Plateau and the Hengduan Mountains. The centroid migration results show an overall shift of the suitable habitat of high-altitude-distributed *H. vulgaris* towards higher latitudes and altitudes. Compared to the current period, the centroid in the 2050s will shift 343.87 km northwestward from Delingha City in Haixi, Qinghai, to a higher latitude. In the 2070s, the centroid will shift to Golmud City in Haixi, moving 431.44 km southwestward (Figure 7 and Figure 8).

**Figure 9:**
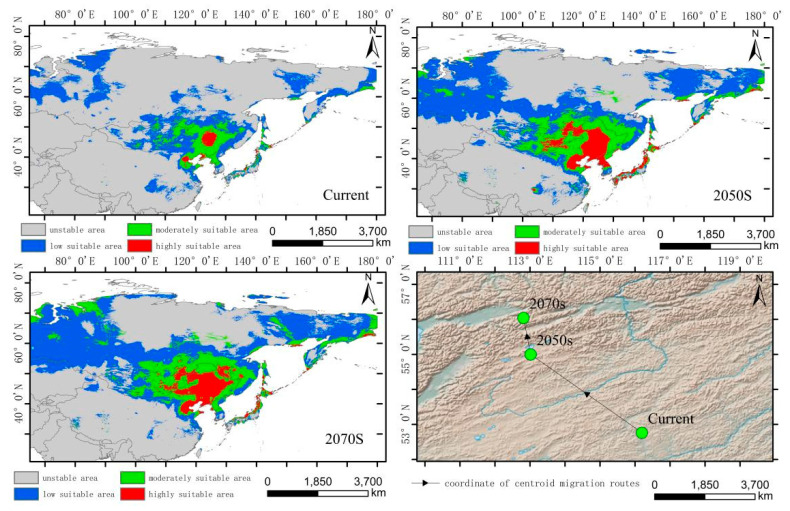
Distribution of suitable areas and centroid transfer of *H. vulgaris* in different periods in low-altitude distribution model.

**Figure 10:**
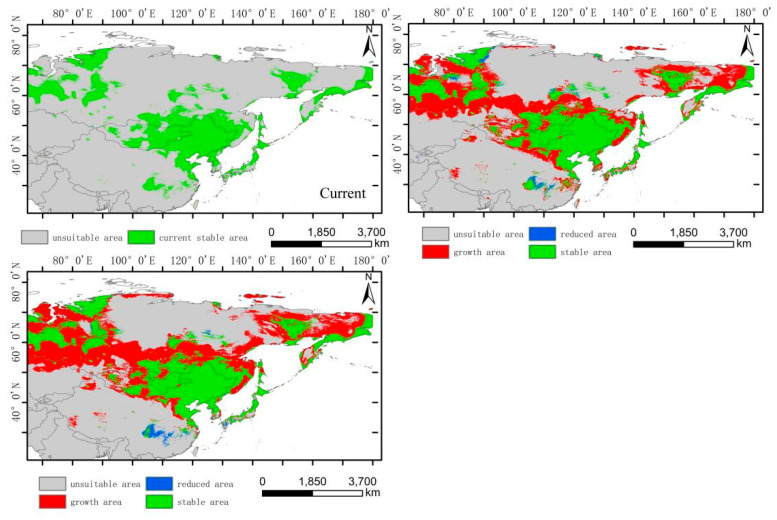
Dynamic changes in suitable area of *H. vulgaris* in different periods in low-altitude distribution model.

The low-altitude-distributed *H. vulgaris* is currently mainly concentrated in the Northeast China Plain, the northern part of the North China Plain, and low-altitude areas of Daxing Anling, Mongolian Plateau, and Inner Mongolia Plateau, as well as the West Siberian Plain and North Siberian Plain in Russia. Comparing the suitable habitat areas and distributions between the current and future periods reveals that the suitable habitat area for low-altitude-distributed *H. vulgaris* will increase significantly with climate change. The suitable habitat area will increase by 99.71% in the 2050s and by 13.29% in the 2070s (Figure 6). The increased areas will mainly expand sharply around the current distribution areas, with substantial growth in high-, medium-, and low-suitability zones, especially the high-suitability zone, which will expand by about 4.37 times. This expansion mainly extends eastward from the northeast low-altitude areas to the Changbai Mountains, the northern part of the Korean Peninsula, and the western part of the Japanese archipelago; westward to the Greater Khingan Range and the Inner Mongolia Plateau; and southward to the northern part of the North China low-altitude area and the Taihang Mountains. The low-suitability zone mainly expands westward and eastward from the West Siberian low-altitude areas, connecting with the medium- and high-suitability zones in Central Asia, forming a continuous area. The expansion trend indicates that by the 2070s, low-altitude-distributed *H. vulgaris* will gradually migrate to higher-altitude areas. The centroid migration results also show an overall shift of the suitable habitat of low-altitude-distributed *H. vulgaris* towards higher latitudes and altitudes. Compared to the current period, the distribution center of *H. vulgaris* will continuously move northwestward from the southwest of Hulunbuir in Inner Mongolia. In the 2050s, it will move 550.32 km northwestward, and in the 2070s, it will move 206.22 km northward (Figure 9 and Figure 10).

**Figure 11:**
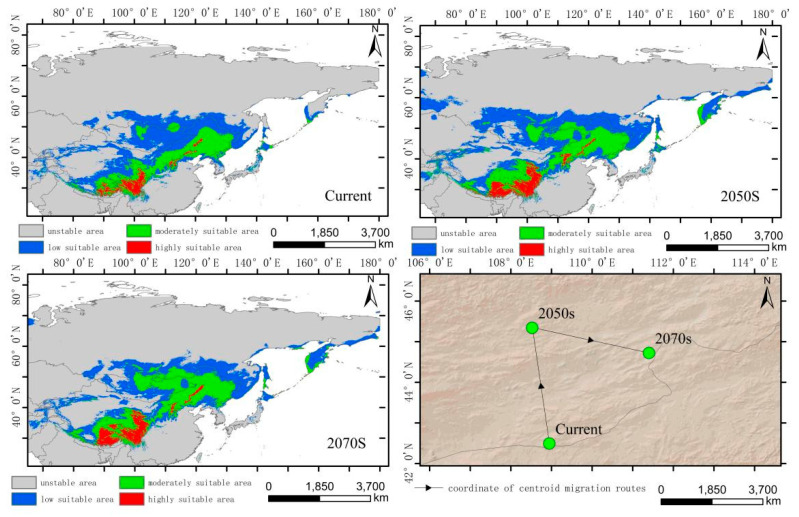
Distribution of suitable areas and centroid transfer of *H. vulgaris* in different periods in integrated distribution model.

**Figure 12:**
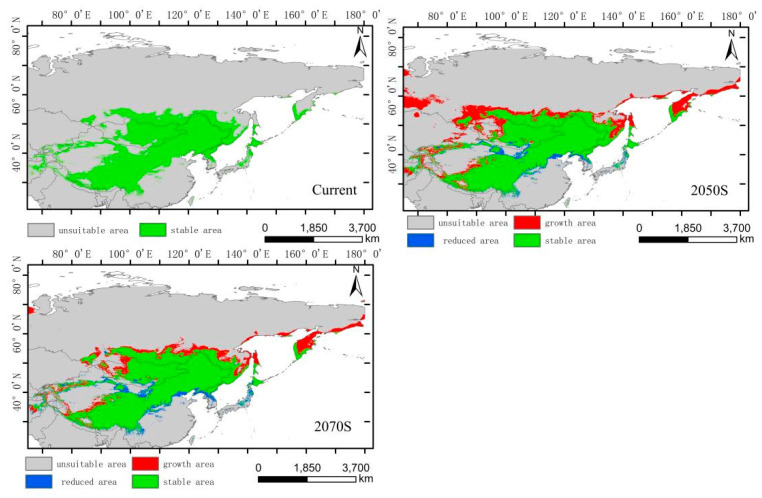
Dynamic changes in suitable area of *H. vulgaris* in different periods in integrated distribution model.

The integrated distribution model indicates that the current potential distribution of *H. vulgaris* is primarily concentrated in the central part of the Asian continent, within the range of 75°–142° E and 27°–56° N. Comparing the suitable habitat areas and distributions between the current and future periods reveals that the trend in the suitable habitat area change and suitability value for the integrated distribution of *H. vulgaris* is consistent with that of the high-altitude distribution, with an increase in the 2050s and a decrease in the 2070s (Figure 6). In the 2050s, the high-, medium-, and low-suitability zones all increase, characterized by expansion towards higher latitudes. In the 2070s, the low-suitability zone significantly decreases. The high-suitability zones for *H. vulgaris* are concentrated in the southwestern part of the Tibetan Plateau and the southeastern Hengduan Mountains. The increased areas shift towards higher latitudes and altitudes. The centroid migration results show an overall shift of the suitable habitat for *H. vulgaris* towards higher northern latitudes. Compared to the current period, the distribution center of *H. vulgaris* will move 443.60 km northwestward in the 2050s from Ordos in Inner Mongolia, and 337.20 km southeastward in the 2070s (Figure 11 and Figure 12).

## 4. Discussion

### 4.1. Differences in Crucial Bioclimatic Variables Across Various Models

In freshwater habitats, climatic variables are fundamental controlling factors for physiological metabolism and the rates of biological processes, determining the distribution and abundance of aquatic plants [36]. Among the three models, temperature factors were the most important environmental factors determining the distribution pattern of *H. vulgaris*, followed by precipitation factors. This is similar to findings from studies of terrestrial and wetland herbaceous plants such as *Solanum Nigrum* [37], *Corydalis* species [38], and *Alternanthera Philoxeroides* [39]. In the integrated distribution model, the environmental variable with the highest contribution rate was the annual mean temperature, with suitable values ranging from −2.12 °C to 9.72 °C, reflecting its adaptation to cold environments. The precipitation in the wettest month and the precipitation in the coldest quarter are climatic factors reflecting water requirements under humid–hot and dry–cold conditions. This indicates that precipitation conditions influence the hydrological conditions of wetlands, and varying amounts of seasonal rainfall recharge can maintain the suitable water levels required by *H. vulgaris*. This is consistent with the ecological characteristics of *H. vulgaris*, which is relatively small in stature, is only suitable for growth in shallow water areas such as marshes, wetlands, lakes, streams, and the banks of rivers [21], and is extremely vulnerable to fluctuations in water levels [40,41]. In the integrated distribution model, the presence of two suitable rainfall ranges in the coldest season suggested that *H. vulgaris* has undergone niche differentiation across different regions.

Plants can adjust their ecological strategies to adapt to different climatic conditions, which is reflected in the significant altitudinal dependence of ecological traits in species with broad altitudinal distributions [42]. The threshold values of crucial bioclimatic variables influencing the distribution of *H. vulgaris* showed significant differences between the two altitudinal populations. In both the high-altitude and low-altitude distribution models, the highest contributing biocliamtic variable was isothermality. A higher isothermality indicates larger diurnal temperature ranges and smaller annual temperature ranges [43]. The suitable value range of *H. vulgaris* in the high-altitude distribution area was significantly higher than that of the low-altitude population and was close to that of the Tibetan Plateau endemic species *Swertia przewalskii* [44]. This suggests that high-altitude and low-altitude populations of *H. vulgaris* have undergone intraspecific differentiation to adapt to different climatic conditions, with the low-altitude population adapting to regions with larger annual temperature ranges and smaller diurnal temperature differences.

In addition to isothermality, the other two dominant bioclimatic variables differed between the two models. For the high-altitude distribution model, they were annual mean temperature and precipitation in the coldest quarter, while for the low-altitude distribution model, they were mean temperature in the driest quarter and precipitation in the wettest quarter. The suitable annual mean temperature range for high-altitude-distributed *H. vulgaris* was slightly lower than that in the integrated distribution model, indicating a stronger adaptation to cold climates [17]. The distribution area of high-altitude-distributed *H. vulgaris* is mainly characterized by alpine plateau climates, with arid and low-rainfall conditions. During the dry and cold season, *H. vulgaris* relies on a certain amount of rainfall for water supply, making precipitation in the coldest quarter a limiting factor for high-altitude-distributed *H. vulgaris*. The distribution area of low-altitude-distributed *H. vulgaris* mostly ranges within a temperate continental climate and temperate monsoon zones, characterized by an extreme annual temperature range, cold and dry winters, and scarce precipitation. Although *H. vulgaris* overwinters in the form of underground rhizomes, long and extremely cold winters mean that the mean temperature of the driest quarter remains a key factor influencing its distribution. In contrast, during the wettest summer months, when precipitation is concentrated, the small stature of *H. vulgaris* leads to its gradual death in deep water areas [40,41]. Therefore, precipitation in the wettest month is an important factor affecting its distribution.

### 4.2. Distribution Pattern and Trends of Suitable Habitats

The results from the integrated altitude distribution model and high-altitude distribution model indicated that potential suitable habitats of *H. vulgaris* initially expanded and then contracted under the climate change scenarios, with populations primarily migrating towards high-altitude regions. The high-suitability areas were all located to the west and north of the Hu Huanyong Line (Heihe-Tengchong Line). Many studies have shown that under global climate change, alpine species are shrinking their habitat and migrating to higher altitudes [11,45,46]. The potential suitable areas of *Carex moorcroftii*, *Stipa purpurea*, *Meconopsis punicea*, and most endemic wetland plants on the Qinghai–Tibet Plateau have decreased [10,47,48]. The distribution changes of 171 forest species in Western Europe over the past century indicate that climate warming has led to an average increase of 29 m per decade in the optimal altitude of species [49]. *H. vulgaris* responds to climate change in a manner consistent with these plants, expanding their ecological niches upwards to track their climatic niches [50,51]. In the 2050s, the expansion of the distribution range of *H. vulgaris* in high-altitude areas is likely to be closely associated with the enlargement of lake surfaces in these regions [52] and the upward shift of the ecological niche suitable for the growth of aquatic plants. As climate warming persists and progresses over time, the distribution of *H. vulgaris* in high-altitude regions is expected to gradually contract due to the uneven spatiotemporal distribution of increased precipitation trends at high altitudes [53] and limitations in colonization space [8].

In contrast, *H. vulgaris* in the low-altitude distribution areas showed a significant expansion trend, with populations continuously migrating towards higher latitudes. Current studies show that in the mid- and high-latitude regions of the Northern Hemisphere, the area of aquatic vegetation in lakes has shown a significant expansion trend over the past 40 years [54]. Under future climate scenarios, the distribution area of aquatic plants may also increase significantly, the suitable habitat areas of *Hydrocharis dubia* and *Hydrocharis morsus-ranae* are projected to significantly increase and shift towards higher latitudes [55], and *Elodea densa*, *Myriophyllum aquaticum*, and *Ludwigia* spp. are anticipated to expand their invasive ranges as their distribution moves toward higher latitudes in Europe and North America [56]. This expansion trend may be due to the increased suitable habitat for plants in high-latitude regions under future climate warming conditions [57].

## 5. Conclusions

In this study, populations of *H. vulgaris* on the Asian continent are distinguished into high-altitude and low-altitude distribution groups, and independent test datasets are used to model each population separately, with the aim of more accurately predicting the species’ distribution across different geographical regions. The results indicate that there are differences in the key bioclimatic variables and suitable values affecting the distribution of sea buckthorn populations in high- and low-altitude regions. Furthermore, the shifts in suitable habitats for these two populations are anticipated to take entirely opposite directions under future climate change scenarios. Specifically, the migration trends indicate a propensity for these populations to move towards areas of a higher altitude and increased latitude.

In response to the formidable challenges posed by climate change, as well as the intense competition resulting from the altitudinal migration of lowland species to higher elevations, the ecological niche of *H. vulgaris* in high-altitude regions may be further compressed, potentially leading to a further contraction in its distribution range. In these areas, it is highly necessary to closely monitor the dynamics of wetland vegetation and to enhance conservation and restoration efforts regarding *H. vulgaris*. In low-altitude regions, the distribution of *H. vulgaris* may undergo significant expansion. It is crucial to focus on monitoring the dynamics of wetland vegetation in low-altitude areas with high latitudes and to enhance the surveillance and management of *H. vulgaris*.

## Figures and Tables

**Table 1:** 19 bioclimatic variables.

Environment Variables	Description	Unit
bio1	Annual Mean Temperature	°C
bio2	Mean Diurnal Range (Mean of monthly (max temp–min temp))	°C
bio3	Isothermality	%
bio4	Temperature Seasonality	-
bio5	Max Temperature of Warmest Month	°C
bio6	Min Temperature of Coldest Month	°C
bio7	Temperature Annual Range	°C
bio8	Mean Temperature of Wettest Quarter	°C
bio9	Mean Temperature of Driest Quarter	°C
bio10	Mean Temperature of Warmest Quarter	°C
bio11	Mean Temperature of Coldest Quarter	°C
bio12	Annual Precipitation	mm
bio13	Precipitation of Wettest Month	mm
bio14	Precipitation of Driest Month	mm
bio15	Precipitation Seasonality (Coefficient of Variation)	-
bio16	Precipitation of Wettest Quarter	mm
bio17	Precipitation of Driest Quarter	mm
bio18	Precipitation of Warmest Quarter	mm
bio19	Precipitation of Coldest Quarter	mm

## Data Availability

Data are contained within the article.
